# The History of Medicine in Bristol and of the Bristol Medico-Chirurgical Society
*An address to the Bristol Medico-Chirurgical Society.


**Published:** 1974-07

**Authors:** C. Bruce Perry

**Affiliations:** Professor Emeritus of Medicine, University of Bristol


### The History of Medicine in Bristol and of the Bristol Medico-Chirurgical Society*

C. Bruce Perry, M.D., F.R.C.P.

Professor Emeritus of Medicine, University of Bristol

In view of the fact that we last year celebrated the 600th anniversary of the granting of Bristol's great Charter, I feel it would be proper to start any review of the history of Medicine in Bristol at the Fourteenth Century.

**The XIVth to XVIIth Centuries**

Unfortunately, we know very little about the organisation or practice of medicine at this time. That great medical historian Dr. Charles Singer wrote of medicine then:— 'The prevailing conception of disease was archaic, diagnosis and prognosis was based on the state of the stars and an inspection of the urine, treatment consisted of blood letting and the use of herbs whose nature was ill understood (perhaps not so different from today if one substitutes drugs for herbs). Anatomy was taught from ancient texts or at best from animal dissection, alchemy was directed towards the elixir of life and surgery was of the meddlesome type'.

A past President of this Society, Dr. George Parker who made a close study of early medical matters in Bristol concluded that records bearing on them are extremely scanty and such facts as we do get are incomprehensible unless compared with what existed elsewhere.

In the XIVth Century probably the majority of orthodox practitioners belonged to the priesthood although this was gradually changing as the church tended to frown on it. Two hundred years before monks had been forbidden to practise medicine outside the monastery. Further, the Church finally forbad priests to shed blood which caused much difficulty when the commonest therapy was venesection. They therefore taught the Barber-surgeons to do this for them. The first record of a Guild of Barber-surgeons in Bristol is contained in the famous Little Red Book under the date of 1395 when they took part in a civic procession. A Bristol Guild was almost certainly in existence before this as the Act of Parliament authorising the Union of Barbers and Surgeons was passed in 1363. The Bristol Guild presumably had a Hall but its site is unkown. In 1690 they built a new hall facing on Shanon's Court and Exchange Avenue. At the dissolution of the Guild in 1745, when the Barbers and Surgeons parted company, the hall became The West India Coffee House and survived until the early years of this century as a public house known as the Grapes with the Grotto restaurant on the ground floor.

Most surgeons in the XIVth century gained their experience in the wars and presumably served a seven year apprenticeship. Guy de Chaillac who mainly practised in Avignon, wrote a treatise on Surgery, a copy of which is in the City Library. An appendix at the end of the Bristol copy reveals something of the state of medicine at that time and reads "Diseases are said to be of two kinds, chronic and acute. Those of a chronic nature are ruled by the movements of the sun, and those of an acute nature by the movements of the moon".

Guy de Chaillac's great contribution was not, however, his book on surgery but his suggestion that a rope should be suspended over a patient's bed so that he could move his position himself. A suggestion for which thousands and thousands of patients must have been grateful.

There were, of course, physicians in the XIVth century. They were said to be learned, scarce and expensive. They were licensed from the Universities of Oxford and Cambridge or from Universities on the continent like Montpeilier, Paris, Padua and Bologna. Their training was entirely theoretical however and consisted of debating and defending the writings of Hippocrates and Galen and the various glosses which had been written on them. The authority of Galen reigned supreme. When Jacobus Sylvius started dissecting the human body and found it not as Galen said he concluded that the Almightly had changed the structure of man since Galen's time. It was not until the XVIth century that this authority began to be questioned and Paracelcus prefaced his lectures at Basel by publicly burning the works of Galen. As late as 1559 Dr. John Geynes was cited before the College of Physicians for impugning the infallibility of Galen on 22 counts and only on his humble recantation signed with his own hand was he absolved from heresy and received back into College. However, he was appointed Censor next year. The most famous physician in England in the XIVth century was John of Gaddesden believed to be the prototype of Chaucer's physician:—

"No one alive could talk as well as he did
On points of medicine and of surgery
For being founded in Astronomy
He watched his patient's favourable star
And by his natural magic knew what are
The lucky hours and planetary degrees
For making charms and magic effigies.
He was a perfect practising physician
Yet he was rather close as to expenses
And kept the gold he won in pestilences
Gold stimulates the heart so we are told
He therefore had a special love of gold."

* An address to the Bristol Medico-Chirurgical Society.

John of Gaddesden wrote a book on medicine which he called the Rosa anglica or Rosa medicinae. He gave us one of the reasons for this title that "As the rose overtops all flowers so this book overtops all treatises on the practice of medicine and it is written for both poor and rich, surgeon and physician so that there shall be no need for them to be always running to consult other books for here they will find plenty about all curable disease both from the special and the general point of view."

All his contemporaries did not take the same view. Guy de Chaillac for instance described it as a foolish English rose containing no sweetness, only a few old fables.

Then there must have been apothecaries who were gradually separating from the grocers. At first only supplying drugs ordered by the physicians they gradually began to prescribe themselves. They too would serve a seven year apprenticeship. This sort of thing persisted until the Medical Act of 1858. The great Edward Jenner was apprenticed at the age of fourteen to Mr. Ludlow of Sodbury. Chaucer in describing his physician said:

> "All his apothecaries in a tribe
> Were ready with a drug he would prescribe
> And each made money from the others guile
> "All his apothecaries in a tribe

By the XVIIIth century, however, the profession was becoming rather more organised. Dr. Harsent in his presidential address to this society in 1899 described four classes of medical practitioners in Bristol in the XVIIIth century.

(1) Physicians who held their heads very high.

They all had a good knowledge of the classics. Quite frequently they never saw their patients but sitting in a coffee house would receive the Apothecary, discuss the case with him and then prescribe. When they did visit a patient it was usually when he was **in extremis** and then only to administer musk and close the eyes of the patient.

Dr. Logan, one of the first physicians apointed to the Infirmary was a strict observer of professional costume never venturing abroad unless in full dress . . . . his head covered by the immense flowing wig of George II's time, a red roquelaire hanging from his shoulders to his heels, his wrist graced with a gold headed cane and his side furnished with a long French rapier.

(2) Surgeons mainly occupied in bleeding patients at the request of the physicians. Such operations as they did carry out were often announced in the lay press, for instance in the Bristol Intelligence for May 1750 we read that "within these few weeks past four boys have been cut for the stone in the Bristol Infirmary by Mr. Thornhill and are all recovered."

(3) There were still some Barber-Surgeons and the first meeting of the Governors of the Infirmary was held in the Barber-Surgeons Hall. It is claimed that the last remnant of Barber surgery in Bristol disappeared with "Old Parsley" who practised as late as 1807 next to the Guildhall in Broad Street and it was said he had dressed more wigs, drew more teeth and spilled more blood than any man in Bristol.

(4) Apothecaries who did most of the "general practice" in the city. Although only allowed to charge for drugs, and not for advice, these were consumed in such vast quantities that many made a considerable income. Dr. Orr-Ewing in his Presidential address to this society quotes Mr. Brodripp as earning £4,000 a year, quite a sum in those days. In 1754 there were apparently in Bristol 5 physicians, 19 surgeons, 13 barber-surgeons and 29 apothecaries. At the end of the XVIIth and early part of the XVIIIth century two important events happened in Bristol.

**The XVIIIth Century**

Up to the end of the XVIIth century each parish was responsible for the care of its own poor but in 1696 John Carey supported the creation of a common fund and a common workhouse for the whole city. This required a special Act of Parliament which was really the first Poor Law Act. This idea was rapidly followed by other places. It was hoped that the work done by the inmates would produce goods which might be sold to defray expenses, for instance the female inmates of the Bristol workhouse were expected to spin cotton. The governing body consisted of the Mayor, Aldermen and 48 guardians. In 1698 they set up the new Union in the Aldworth mansion which had later been a sugar refinery in which Edward Colston was interested and for the last two years had been the local Mint. This soon became known as St. Peter's Hospital and the building survived as the offices of the Guardians until destroyed by enemy action in 1940. When it was opened Dr. Thomas Dover, the inventor of Dover's powder (Pulv. Ipecac Co.) applied for and was appointed to the post of Honorary Physician to the hospital. It was Dr. Dover who sailed with Woode Rogers on the Duke and Duchess in 1708 on a voyage round the world. Going ashore on the Island of Juan Fernandez Dover found Alexander Selkirk who had been marooned there alone over four years before. Selkirk was brought safely back to England and formed the basis of Defoe's 'Robinson Crusoe'. Dover in his own time was known as The Quick Silver Doctor owing to the fact that he regarded mercury as an infallible panacea for nearly all ills. He fell foul of the College of Physicians however and wrote a book 'The Ancient Physicians' Legacy to This Country' which seems to have been largely an attack on the Fellows. At first St. Peter's Hospital flourished and Dr. Dover insisted on a diet better than that of the average employed labourer. However the building was too antiquated to be really suitable and in 1786 Sir Frederick Eden gave it a bad report referring to filth, bugs and vermin. It became grossly overcrowded and in 1833 had 600 inmates and the aged, infirm and lunatics predominated over those who could work.

The other important medical event in Bristol in the XVIIIth century was the establishment of the Infirmary. Towards the close of the year 1736 some well disposed persons had a meeting at which they resolved to endeavour at the establishment of a public charity "since many sick persons languish and die miserably for want of necessaries" and "desiring as far as in us lies to find some remedy for this great misery of our poor neighbours" they resolved to subscribe to the "procuring, furnishing and defraying the necessary expense of an Infirmary at Bristol". This memorandum was signed by 78 persons promising sums of from two to six guineas. Thus was founded the Infirmary.

After much discussion as to the best site a lease was taken of a "Loft, warehouse, cellar and other buildings and waste land previously used as a brewery situate in Maudlin Lane". John Ellbridge was appointed Treasurer, a wealthy man, Controller of His Majesty's Customs he contributed at least £1,500 to the Infirmary in the first two years of its existence. On May 20th 1739, the first staff were appointed:— 4 physicians, 2 surgeons and an apothecary. The senior physician was Dr. John Bonython whose services are commemorated on the same board as that recording Ellbridge's benefactions. The building was opened for out-patients on June 20th, 1737 and the first in-patients—17 men and 17 women were admitted on December, 13th 1737. After the formal opening there was a procession to St. James Church where a sermon was preached. The company then repaired to the Nag's Head Tavern in Wine Street where they dined together finishing the day amidst the smoke of tobacco and emptying and replenishing mugs of fat Bristol ale. This dinner was an annual event until 1780 when it ceased because the proceedings were apt to become rather violent. The affairs of the Infirmary were sometimes discussed with such animation "that broken heads and bloody noses resulted".

House visitors were appointed from the opening and at the second meeting on December, 19th, 1737 they examined the beer and "found it not good enough".

In 1742 a plan was drawn up for a building with two wings and by 1755 the number of beds had been increased to 134. However, in 1782 a general meeting was held to consider an entirely new building. The Faculty — as the medical staff were called — wanted to build on the Red Lodge Site — then of course surrounded by gardens with a superb view over the city. But despite this it was decided to rebuild on the old site. This was done in stages during which the building was turned round so that the entrance instead of being on the south side was on the north. The east block was ready for occupation in 1786, the centre block was completed six years later and the west wing in 1811. It could now accommodate 180 patients.

**The XIXth Century**

However, it soon became clear that despite this the Infirmary could not accommodate all those needing its help. In 1831 a committee was formed of benevolent persons, many of whom belonged to the Society of Friends, "whose minds recoiled from the reflection that in a City so assuredly charitable and wealthy, numbers of their fellow creatures were hourly pining under the visitation of disease" to decide whether other means could be devised for the mitigation of the miseries they deplored.

1831 was not a very auspicious year for such a venture. Grave financial depression had followed the Napoleonic wars, there had been riots all over the country and in Bristol on October 29th one side of Queen Square was burnt and the Bishop's Palace wrecked. In the following year Bristol experienced a serious outbreak of cholera with a total of 1612 reported cases with 626 deaths. However the committee persevered and the General Hospital was opened in some houses in Guinea Street on November 1st, 1832. The medical staff appointed by vote at a public meeting held at St. Peter's Hospital, the previous December consisted of 3 physicians and 4 surgeons. Perhaps the most distinguished of the first physicians was John Addington Symonds who was Lecturer at the Royal College of Physicians in 1858 and President of the British Medical Association in 1863. He conducted a large practice from Clifton Hill House and was the father of John Addington Symonds junior the Victorian literary critic and author of the seven volume work on the Italian Renaissance. For some years the hospital was in considerable financial difficulties but gradually things improved. An appeal for a new building was launched in 1850 but it was eight years before the patients were transferred to the new building on August, 3rd, 1858. Thereafter the Infirmary and General Hospital continued to care for the sick of Bristol in perhaps not too friendly rivalry. In 1850 the title Royal was granted to the Infirmary which was largely supported by the Church of England and Tories, whereas the Hospital supporters were largely non-conformists and Whigs. Hence the saying that patients could expect a sovereign remedy at the Infirmary but a radical cure at the Hospital.

From time to time additions were made to the two hospitals but the major building at the Royal Infirmary was the King Edward VII memorial wing completed in 1913 and at the General Hospital two new wards and accommodation for residents in 1914 and a new out-patients department and two further wards in 1932.

**Medical Education**

From the first the Apothecary at the Infirmary was allowed apprentices and shortly afterwards surgical apprentices were admitted. These were of two types, either they were attached to an individual surgeon and lived in his house, as Jenner did with John Hunter or they were Infirmary pupils attached to all the surgeons. Thus began medical education in Bristol. The Infirmary physicians were for some unexplained reasons not allowed pupils until 1832. Students were admitted to the General Hospital from the start, in fact the statement of policy made at the opening ceremony stated "your committee cannot doubt that your Establishment in its progress will become a useful School of Medical Instruction". Various lectures on anatomy had from time to time been organised by members of the staff of the Infirmary. For instance Page and Ford gave lectures in the anatomical theatre of the Barber-Surgeons Hall in 1744-1746. Beddoes of the Pneumatic Medical Institution fame started a course at Red Lodge in 1797. He arrived in Bristol in 1793 having retired from a Readership in Chemistry at Oxford on account of unpopularity through expressing sympathy with the French revolutionaries. He developed the Pneumatic Medical Institute in Dowry Square which is perhaps mainly famous as giving Humphrey Davy his start when he was appointed Superintendent of the Institute. Here he experimented with Nitrous Oxide on himself, Coleridge, Southey and various lady friends. It is surprising that there were no tragedies but as you know he survived and left Bristol for the Royal Institution in 1801. One of Beddoes' more bizarre therapies was the treatment of consumption with cow's breath. The cow was taken into the bedroom and its muzzle inserted between the bed curtains. But to return to Medical Education. Perhaps we should remember him as one of the earliest exponents of preventive medicine as he wrote a book entitled "A guide to self preservation and parental affection, or plain directions for enabling people to keep themselves and their children free from several common disorders".

The Royal College of Surgeons obtained its charter in 1800 and its examinations while not compulsory became popular. In 1815 the Society of Apothecaries was authorised to prosecute unqualified apothecaries and to examine and license all new men. So medical education was becoming more formalised. Repeated efforts were made by the staffs of the two Bristol Hospitals to provide regular courses of instruction. Mr. Shute in 1807 built dissecting rooms in Limekiln Lane, Lower College Street and this became known as the Bristol School of Anatomy and Medicine. In 1812 Francis Gold lectured on Anatomy and Physiology at 3, College Green, near the cloisters — a spot now covered by the nave of the Cathedral. This Francis Gold had been a prisoner in Paris during the Napoleonic wars and had been released by Napoleon in response to a letter from Jenner, Napoleon saying he could refuse nothing to so great a man. In 1826 Dr. Henry Clarke started another school at 3, King Square which he called the Bristol Medical Surgical School.

After much discussion all these schools combined in 1833 to form the Bristol Medical School moving to its new buildings in Old Park—now known as Lunsford House—in 1834. The seal of the Medical School is rather mysteriously dated 1828. This is supposed to be because this was the date when the Society of Apothecaries first recognised lectures given in Bristol. The school flourished and within two years the Apothecaries found it the largest provincial medical school after Manchester and Birmingham. In 1873 it was proposed that instead of raising funds for a new building the school should join forces with proposed College of Literature and Science for the West of England. This was agreed and the medical school joined University College when it was founded in 1876. However, despite the fact that the Medical School did so much to help the foundation of the College there was much discussion as to the actual nature of the union.

Finally, in 1879 the Medical School became affiliated with University College but with its own governing body. This decision may have been hastened by attacks on the school being made by the Lancet because of the high failure rate of Bristol students in the College of Surgeons examinations. In 1892 a new medical wing of University College was opened by Sir Andrew Clarke. This now houses the Department of Geography. Having managed to work together happily for more than twelve years, full incorporation was decided on in 1893 and in this year the Medical School became the Medical Faculty of University College with Dr. Markham Skerrett as first Dean. No doubt in recognition of the part played by the Medical School in the development of the College when the University received its charter in 1909 Dr. Michell Clarke — the Professor of Medicine — was appointed Pro-Vice-Chancellor which post he held till his death in 1918.

Despite the amalgamation of these two the clinical teaching remained a proprietary school in the hands of the Staffs of the B.R.I. and B.G.H. to whom the University handed over all clinical fees. In 1922 the clinical teachers surrendered their rights to the University. Hence arose the Clinical Board of the University of which all clinical teachers were members and the Ordnance establishing which was only rescinded by Court in 1964.

The British Medical Association founded as the Provincial Medical and Surgical Association in 1832 held its first Anniversary meeting in Bristol in 1833. The meeting occupied one day and the company dined at six o'clock. It is recorded that nothing could exceed the harmony and conviviality of the party . . . . the company did not rise until a late hour . . . . the turtle, dessert and wine did the hotel the highest credit. The Association met in Bristol again in 1863 and 1894. This meeting much resembled the annual meeting of today. It was divided into sections and there was much social activity—garden parties, luncheons, receptions, dinners, concerts and balls.

In 1875 the British Association met in Bristol and it had been hoped that Dr. Beddoe who had recently retired as Physician to the Infirmary and was a world renowned anthropologist would be President of the Anthropological section. However, he refused this honour as follows:— "I could not afford to let my scientific reputation injure my medical practice."

The other outstanding figure in medicine in Bristol in the XIXth century was William Budd. In his book on Typhoid in 1873 he first put forward the view that typhoid was spread by the faeces. In 1876 he suggested that phthisis was contagious—15 years before Koch described the Bacillus tuberculosis—as follows:— "The idea that phthisis is a self-propagated disease and that all the leading phenomena of its distribution may be explained by supposing that it is disseminated through society by specific germs contained in the tuberculous matter cast off by persons already suffering from the disease, first came to my mind, unbidden so to speak, while I was walking on the Observatory Hill at Clifton in the second week in August, 1856". He waited 11 years before publishing this idea.

It is interesting that despite this and Koch's discovery of the Bacillus in 1882, the Society's journal for 1883 contained two papers. One is entitled "Proofs of the Existence of the Phthisical Contagion" by Dr. Shingleton Smith and the other "Clinical Evidence against the Contagiousness of Phthisis" by Dr. Markham Skerrett—President in 1892.

Perhaps the most famous member of the Bristol Medical School in the latter half of the XIX century was W. G. Grace who entered the school as a student in 1868 and finally qualified eleven years later via Westminster and St. Bartholomew's.

#### Bristol Medico-Chirurgical Society

From time to time various starts had been made with a medical society in Bristol. One founded in 1788 met for some years at the Infirmary, a Medical and Chirurgical Society started in 1812 had a monthly supper and circulated books and lasted for about 12 years. Then in 1819 a Medical and Chirurgical Association was formed with ethical aims, it decided on a scale of fees and dined twice a year but this only lasted a few years. There is then a gap until February 24th, 1874 when a meeting of practitioners in Bristol was called by Mr. Nelson Dobson, Dr. Shingleton Smith and Dr. Spencer to consider the establishment "for Bristol and Clifton of a new medical society the prominent feature of which will be the development of Pathological and Clinical resources of the place, but formed on a basis sufficiently broad to include all the departments of Scientific and Practical Medicine." The meeting was attended by 29. Letters in support were received from six and verbal support from a further nine. Thus was founded our Society. It progressed rapidly. In less than two weeks rules had been formulated by the committee and the first ordinary meeting was held on March 25th just after a month from the date of its inception. The early meetings were largely devoted to the report of cases and demonstrations of pathological specimens. But in June, 1876 a discussion on Scarlet Fever took place which was opened by Dr. Brittain, the first President, and this aroused so much interest that the discussion was continued at a meeting in September. In the following May Dr. Shingleton Smith, one of the promoters of the Society and President in 1888, opened a discussion on "Treatment of High Temperatures of the Body". This discussion was continued at both the September and November meetings.

The new society immediately flourished and in view of the funds it was decided in 1876 "that no subscription for the year 1877 should be required of old members." In 1878 it was resolved to publish Transactions of the Society and the first volume appeared that year. Although further issues had been planned none appeared but in 1882 it was decided that "In the opinion of the Society the time has come when the various medical and surgical interests of the West of England ought to be united by the publication of a periodical journal which might appear under the name of the Bristol Medico-Chirurgical Society . . . . it to be called the Bristol Medico-Chirurgical Journal." As you know the journal has continued ever since but has had a rather difficult financial career. In its second year the Society decided that in view of the exceptional circumstances as regards expenses of publication of the journal, no contribution should be voted to the Medical Benevolent Fund. Ten years later however, it was possible to vote £100 from the Journal funds for the purchase of books. The financial problems of the journal continue and as many of you will remember the title was changed in 1952 in an attempt to increase the circulation, to the Medical Journal of the South West. This had little or no effect and nine years ago the journal reverted to the original title.

The Society can confidently claim that throughout its hundred years it has continued as its founders hoped, to promote the Pathological and Clinical resources of the place. It has always been quick to bring medical advances to the notice of its members. In 1894 an oxy-hydrogen lantern was purchased and lantern slides were shown for the first time. In the same year a sphygmogram was demonstrated illustrating a "pulsus bisferiens". A whole evening was devoted to the use of anti-toxin in Diphtheria. In 1897 the Society was shown a radiograph made by Roentgen's Rays of a needle in a lady's wrist by Professor Wertheimer of the Merchant Venturers' Technical College. In 1900 Dr. J. G. Swayne read a paper on Ergot of Rye as an oxytocic "which the German writers called the English accoucheur's Herr Gott". At the November meeting of the same year Mr. Paul Bush showed upwards of 200 lantern slides of hospital and other scenes taken during the Boer War. In 1906 Dr. Walter Chase of Boston gave a demonstration by the cinematograph of epilepsy and other nervous diseases. In 1909 a meeting devoted to milk resulted in a committee being set up to consider the question of public action with reference to the City Milk Supply.

The Society has always been intrested in both undergraduate and post-graduate education and in 1888 the committee resolved:—

(1) that a library is desirable.
(2) that it should be in conjunction with the Medical School.

By 1890 many periodicals were being received in exchange for the journal and the need for a reading room became obvious, and a reading room and library were rented from the Scientific and Literary Club at 28, Berkeley Square. Some members, however, thought that the library should be transferred to the New Medical School Buildings as soon as possible but there was opposition to this. Finally Mr. Greig Smith, the Editor of the journal threatened to resign both as editor and as a member unless this were done and in 1892 the library moved to the medical school. At first it was housed in a large room on the first floor but in 1911 the University notified the society that the library was being converted to a Council Chamber. The library was then moved to accommodation in the Old Blind Asylum and then, when the construction of the Wills Memorial Buildings started, to the racquet courts of the old drill hall. With the opening of the new building the library moved to its new site with a room for the society in the basement. There it remained for 24 years. Relationships between the Society and the University were not always happy. In 1915 the University was charging the society 50 guineas a year rent which the Society said it could not pay and a meeting was held between representatives of the Society and the Finance Committee of the University. The Society's representatives reported back that the meeting had been devoid of any result except professions of sympathy on the part of the University at the poverty of the Society. In fact in 1917 it was resolved "That the Agreement between the University . . . and the Bristol Medico-Chirurgical Society . . . . be determined forthwith as the financial position of the Society renders it impossible to provide the rental". However, wiser considerations prevailed. By 1921 the committee proposed that an approach should be made to the University to ascertain if it would wish to accept the Medico-Chirurgical Society library . . . . on conditions mutually agreeable. There were prolonged negotiations and the agreement was finally signed in 1926. With the completion of the new medical school buildings in 1959 a new agreement was made whereby the Society relinquished the use of the room in the University but should have the right to hold the meetings in the medical school, should have the use of a committee room when needed and all members should enjoy membership of the medical senior common room.

In 1908 a long discussion took place on the admission of women to the society and it was decided that nothing in the rules prevented women being elected. However, it was not until 1912 that Dr. Lily Baker was elected to the society as the first woman member and in 1951 the Society elected its first woman President —Dr. Victoria Tryon.

From time to time the Society has held dinners. In 1913 when Dr. Shingleton Smith retired as Editor of the journal after 20 years he was given a dinner consisting of eight courses followed by coffee and accompanied by 13 songs and recitations and concluding with a lantern lecture by Dr. Munro Smith. Amongst those singing were two future presidents of the Society. The Society's first public meeting was held in 1923 when Mr. Howard Somervill, the medical officer of the first Everest expedition gave a lecture illustrated by a film on the "Problems of Living at High Altitudes".

In 1931 Dr. Kenneth Wills that year's President presented the Society with the Presidential badge. As you can see the Society has continued to carry out its objects of promoting the advancement of medical knowledge and has happily steered clear of medical politics. No mention of the agitation occasioned by the introduction of Lloyd George's National Insurance Act appears anywhere in the proceedings of the Society although the Journal does contain one or two sober editorials on it but nothing in the least acrimonious. Similarly at the end of the last war when other bodies were debating whether or not members should accept appointment on committees of the National Health Service this Society merely went on organising meetings and lectures unruffled by the turmoil outside. Some of the more illustrious members of the profession in Bristol in the early part of this century were Dr. Carey Coombs with an international reputation for his work on Rheumatic Heart Disease; Professor Hey Groves who started in general practice in Kingswood became virtual founder and for a long time Editor of the British Journal of Surgery and President of the Society in 1932; Professor J. A. Nixon for many years Editor of the Journal and President in 1922; Patrick Watson Williams starting as Physician at the B.R.I. became one of the Father Founders of Oto-Rhino-Laryngology.

There Mr. President may I conclude this account of the history of medicine in Bristol and of the Society, which for the last hundred years have been really synonymous, by congratulating the Society on the success of its first hundred years and wishing it an even more successful future.

